# 3-Diazo-*N*-[(2*S*)-1-hy­droxy­propan-2-yl]-2-oxopropanamide

**DOI:** 10.1107/S1600536811014413

**Published:** 2011-04-22

**Authors:** Xiao-na Chen, Wen-hao Hu, Xiao-liu Li, Hua-dong Xu

**Affiliations:** a College of Chemistry and Environment Science, Hebei University, 180 East Wu Si Road, Baoding 071002, People’s Republic of China; b Institute of Drug Discovery and Development, and Department of Chemistry, East China Normal University, Shanghai 200062, People’s Republic of China

## Abstract

In the title compound, C_6_H_9_N_3_O_3_, the 3-diazo-2-oxopropan­amide section of the mol­ecule is nearly planar, with a maximum deviation of 0.025 (1) Å from the mean plane of its constituent atoms. The diazo C=N=N angle is 178.0 (3)°. In the crystal, pairs of inter­molecular O—H⋯O and N—H⋯O hydrogen bonds link the mol­ecules into infinite double chains along the [100] direction. The double chains are additionally stabilized by weak C—H⋯O contacts with C⋯O distances of 3.039 (3) Å. Neighboring double chains in turn inter­act with each other through π–π stacking inter­actions [centroid–centroid distance of the 3-diazo-2-oxopropanamide units = 3.66 (6) Å] to form infinite stacks along the *b* axis. Mol­ecules from neighboring stacks inter­digitate with each other in the *c*-axis direction, thus leading to an inter­woven three-dimensional network held together by O—H⋯O, N—H⋯O and C—H⋯O inter­actions and π–π stacking.

## Related literature

For general background to diazo compounds, see: Doyle & Forbes (1998 ▶); Doyle (1986 ▶); Zhang & Wang (2008 ▶). For the synthetic procedure, see: Pedone & Brocchini (2006 ▶).
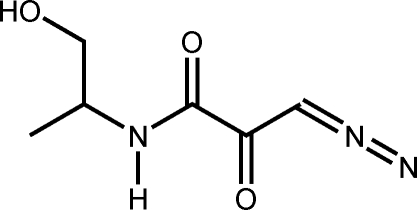


## Experimental

### 

#### Crystal data

C_6_H_9_N_3_O_3_
*M* *_r_* = 171.16
Orthorhombic, 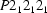

*a* = 5.3136 (3) Å
*b* = 6.7551 (3) Å
*c* = 23.3958 (11) Å
*V* = 839.77 (7) Å^3^
*Z* = 4
Mo *K*α radiation
μ = 0.11 mm^−1^
*T* = 296 K
0.46 × 0.38 × 0.32 mm

#### Data collection

Bruker SMART CCD area-detector diffractometer
Absorption correction: multi-scan (*SADABS*; Sheldrick, 1996 ▶) *T*_min_ = 0.951, *T*_max_ = 0.965
9692 measured reflections
903 independent reflections
826 reflections with *I* > 2σ(*I*)
*R*_int_ = 0.027

#### Refinement

*R*[*F* ^2^ > 2σ(*F* ^2^)] = 0.039
*wR*(*F* ^2^) = 0.123
*S* = 1.17
903 reflections
109 parameters
H-atom parameters constrained
Δρ_max_ = 0.21 e Å^−3^
Δρ_min_ = −0.14 e Å^−3^

### 

Data collection: *SMART* (Bruker, 2007 ▶); cell refinement: *SAINT* (Bruker, 2007 ▶); data reduction: *SAINT*; program(s) used to solve structure: *SHELXS97* (Sheldrick, 2008 ▶); program(s) used to refine structure: *SHELXL97* (Sheldrick, 2008 ▶); molecular graphics: *SHELXTL* (Sheldrick, 2008 ▶); software used to prepare material for publication: *SHELXTL*.

## Supplementary Material

Crystal structure: contains datablocks I, global. DOI: 10.1107/S1600536811014413/zl2366sup1.cif

Structure factors: contains datablocks I. DOI: 10.1107/S1600536811014413/zl2366Isup2.hkl

Additional supplementary materials:  crystallographic information; 3D view; checkCIF report

## Figures and Tables

**Table 1:** Hydrogen-bond geometry (Å, °)

*D*—H⋯*A*	*D*—H	H⋯*A*	*D*⋯*A*	*D*—H⋯*A*
O1—H1*C*⋯O1^i^	0.82	1.99	2.811 (4)	179
N1—H1*D*⋯O2^ii^	0.86	2.35	3.1024 (18)	146
C6—H6*A*⋯O3^iii^	0.93	2.18	3.039 (3)	153

Symmetry codes: (i) 
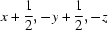
; (ii) 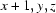; (iii) 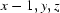.

**Fig. 1.**
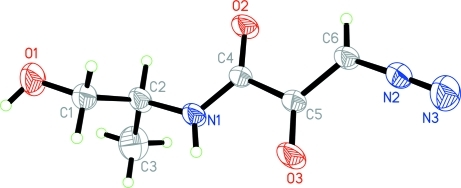
The molecular structure of the title compound, with displacement ellipsoids at the 30% probability level.

**Fig. 2.**
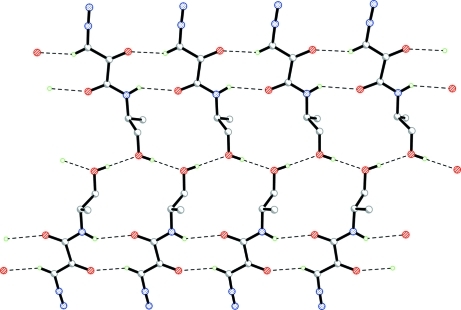
Part of infinite double chains structure of the title compound, linked via hydrogen bonds (dashed lines) extending in the [1 0 0] direction. H atoms have been omitted for clarity, except for those involved in hydrogen-bonding interactions.

**Fig. 3.**
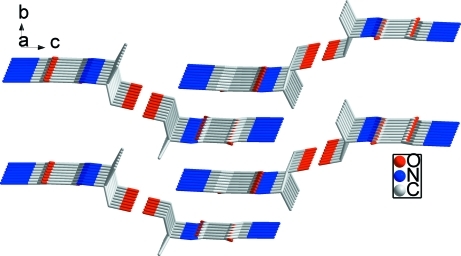
The π–π stacking interactions in the structure of the title compound.

## supplementary crystallographic information

### Comment

Diazo refers to a type of organic compounds that have two linked nitrogen atoms as a terminal functional group. The simplest example of a diazo compound is diazomethane. The electronic structure of diazo compounds involves a positive charge on the central nitrogen and negative charge distributed between the terminal nitrogen and the carbon. The diazo compounds have wide applications in organic synthesis, such as C—H or C—N bonds insertion, 1,3-dipolar cyclization and transition metal complexes catalyzed transformations (Doyle & Forbes, 1998; Zhang & Wang, 2008; Doyle, 1986). To investigate the relationship between structure and reactivity, the title compound was synthesized and its structure was determined by X-ray diffraction. In this article, we present the synthesis and crystal structure of this new diazo compound.

As shown in figure 1, the 3-diazo-2-oxopropanamide section of the molecule is nearly planar with a maximum deviation of 0.025 (1) Å from the mean plane of its constituting atoms. The diazo unit is almost linear with an C5–N2–N3 angle of 178.0 (3)°. The N2—N3, C4—O2 and C5—O3 bond length of 1.113 (3), 1.215 (3) and 1.213 (3) Å, respectively, indicate the presence of a typical N═N and C═O bonds. Whereas the C1–O1 [1.396 (3) Å] and C2–N1 [1.468 (2) Å] bond lengths correspond to typical single bonds.

In the crystal structure, it is noteworthy that pairs of intermolecular O—H···O and N—H···O hydrogen bonds link the molecules into infinite double chains along the [1 0 0] direction. The double chains are further stabilized by weak C—H···O contacts with the C···O distances of 3.039 (3) Å (Fig. 2). Neighboring double chains are in turn interacting with each other through π–π stacking interactions [centroid to centroid distances of the 3-diazo-2-oxopropanamide units are 3.66 (6)Å] to form infinite stacks along *b*. Molecules from neighboring stacks interdigitate with each other in the *c*-direction, thus leading to an interwoven three dimensional network held together by O—H···O, N—H···O and C—H···O interactions and π–π stacking.

### Experimental

To a dried flame-dried 20 ml three-necked round bottomed flask filled with nitrogen and equipped with a refluxing condenser was added diazo ethyl pyruvate (0.5 g, 3.5 mmol), (*S*)-2-aminopropan-1-ol (0.345 g, 4.6 mmol) in 10 ml anhydrous ethanol. This suspension was stirred at room temperature and the reaction was monitored by TLC. When the diazo ethyl pyruvate was consumed, the yellow brown reaction mixture was concentrated to dryness. The crude product was purified by column chromatography on silica gel with petroleum ether/ethyl acetate (1/1) as eluent to give the product in yield of 63% (0.377 g, 2.2 mmol). Single crystals suitable for X-ray diffraction study were obtained by recrystallization of the crude from a diethyl ether solution. ^1^H NMR (CDCl_3_, 400 MHz), δ (p.p.m.): 6.36 (s, 1H), 4.05 (br, 1H), 3.69 (m, 1H), 3.61 (m, 1H), 2.25 (br, 1H), 1.61 (s, 1H), 1.24 (d, *J* = 6.8 Hz, 3H); ^13^C NMR (125 MHz, CDCl_3_), δ (p.p.m.): 181.64, 159.86, 65.38, 54.99, 47.65, 16.42; IR (KBr pellet, ν, cm^-1^): 3327, 3083, 2110, 1733, 1666, 1536, 1381, 788, 706.

### Refinement

All H atoms were placed in idealized positions (C—H = 0.93–0.98 Å, N—H = 0.86 Å, O—H = 0.82 Å) and refined as riding atoms with *U*_iso_(H) = 1.2*U*_eq_(C, N) and with *U*_iso_(H) = 1.5*U*_eq_(O). The methyl H atoms were set based on angle considerations (AFIX 33 instruction in *SHELXL97* (Sheldrick, 2008)). In the absence of significant anomalous scattering effects, 572 Friedel pairs were averaged prior to the final refinement.

### Crystal data

**Table:**

C_6_H_9_N_3_O_3_	*F*(000) = 360
*M**_r_* = 171.16	*D*_x_ = 1.354 Mg m^−^^3^
Orthorhombic, *P*2_1_2_1_2_1_	Mo *K*α radiation, λ = 0.71073 Å
Hall symbol: P 2ac 2ab	Cell parameters from 4465 reflections
*a* = 5.3136 (3) Å	θ = 1.7–25°
*b* = 6.7551 (3) Å	µ = 0.11 mm^−^^1^
*c* = 23.3958 (11) Å	*T* = 296 K
*V* = 839.77 (7) Å^3^	Block, colourless
*Z* = 4	0.46 × 0.38 × 0.32 mm

### Data collection

**Table:**

Bruker SMART CCD area-detector diffractometer	903 independent reflections
Radiation source: sealed tube	826 reflections with *I* > 2σ(*I*)
graphite	*R*_int_ = 0.027
φ and ω scans	θ_max_ = 25.0°, θ_min_ = 1.7°
Absorption correction: multi-scan (*SADABS*; Sheldrick, 1996)	*h* = −6→6
*T*_min_ = 0.951, *T*_max_ = 0.965	*k* = −7→8
9692 measured reflections	*l* = −27→27

### Refinement

**Table:**

Refinement on *F*^2^	Primary atom site location: structure-invariant direct methods
Least-squares matrix: full	Secondary atom site location: difference Fourier map
*R*[*F*^2^ > 2σ(*F*^2^)] = 0.039	Hydrogen site location: inferred from neighbouring sites
*wR*(*F*^2^) = 0.123	H-atom parameters constrained
*S* = 1.17	*w* = 1/[σ^2^(*F*_o_^2^) + (0.0698*P*)^2^ + 0.1238*P*] where *P* = (*F*_o_^2^ + 2*F*_c_^2^)/3
903 reflections	(Δ/σ)_max_ < 0.001
109 parameters	Δρ_max_ = 0.21 e Å^−^^3^
0 restraints	Δρ_min_ = −0.14 e Å^−^^3^

### Special details

**Table:**

Geometry. All e.s.d.'s (except the e.s.d. in the dihedral angle between two l.s. planes) are estimated using the full covariance matrix. The cell e.s.d.'s are taken into account individually in the estimation of e.s.d.'s in distances, angles and torsion angles; correlations between e.s.d.'s in cell parameters are only used when they are defined by crystal symmetry. An approximate (isotropic) treatment of cell e.s.d.'s is used for estimating e.s.d.'s involving l.s. planes.
Refinement. Refinement of *F*^2^ against ALL reflections. The weighted *R*-factor *wR* and goodness of fit *S* are based on *F*^2^, conventional *R*-factors *R* are based on *F*, with *F* set to zero for negative *F*^2^. The threshold expression of *F*^2^ > 2σ(*F*^2^) is used only for calculating *R*-factors(gt) *etc*. and is not relevant to the choice of reflections for refinement. *R*-factors based on *F*^2^ are statistically about twice as large as those based on *F*, and *R*- factors based on ALL data will be even larger.

### Fractional atomic coordinates and isotropic or equivalent isotropic displacement parameters (Å^2^)

**Table:**

	*x*	*y*	*z*	*U*_iso_*/*U*_eq_	
O1	0.4929 (5)	0.2784 (4)	0.01785 (8)	0.0988 (9)	
H1C	0.6387	0.2627	0.0073	0.148*	
O2	−0.0639 (3)	0.5086 (4)	0.16571 (7)	0.0626 (6)	
O3	0.4091 (3)	0.5355 (5)	0.26799 (8)	0.0855 (9)	
N1	0.3555 (4)	0.5279 (4)	0.15304 (8)	0.0617 (7)	
H1D	0.4963	0.5452	0.1705	0.074*	
N2	0.0058 (4)	0.5073 (3)	0.33780 (8)	0.0539 (5)	
N3	0.0377 (5)	0.5040 (5)	0.38481 (9)	0.0801 (8)	
C1	0.4894 (7)	0.3193 (5)	0.07632 (11)	0.0653 (8)	
H1A	0.6612	0.3245	0.0903	0.078*	
H1B	0.4045	0.2121	0.0960	0.078*	
C2	0.3606 (5)	0.5101 (5)	0.09052 (9)	0.0592 (7)	
H2A	0.1870	0.5038	0.0765	0.071*	
C3	0.4882 (9)	0.6869 (6)	0.06378 (14)	0.0911 (11)	
H3A	0.3976	0.8050	0.0736	0.137*	
H3B	0.4904	0.6717	0.0230	0.137*	
H3C	0.6577	0.6963	0.0777	0.137*	
C4	0.1495 (4)	0.5190 (4)	0.18416 (9)	0.0454 (6)	
C5	0.1975 (4)	0.5223 (5)	0.24897 (9)	0.0500 (6)	
C6	−0.0232 (4)	0.5110 (4)	0.28220 (8)	0.0487 (6)	
H6A	−0.1818	0.5063	0.2655	0.058*	

### Atomic displacement parameters (Å^2^)

**Table:**

	*U*^11^	*U*^22^	*U*^33^	*U*^12^	*U*^13^	*U*^23^
O1	0.0713 (13)	0.162 (2)	0.0635 (12)	0.0092 (19)	0.0014 (12)	−0.0457 (14)
O2	0.0318 (8)	0.1035 (15)	0.0526 (9)	−0.0011 (11)	−0.0073 (7)	−0.0031 (11)
O3	0.0278 (8)	0.175 (3)	0.0533 (10)	−0.0017 (14)	−0.0039 (7)	−0.0179 (14)
N1	0.0315 (9)	0.1099 (19)	0.0437 (10)	−0.0016 (15)	−0.0021 (8)	−0.0148 (12)
N2	0.0382 (10)	0.0707 (13)	0.0528 (11)	−0.0018 (13)	0.0038 (9)	−0.0049 (10)
N3	0.0732 (16)	0.115 (2)	0.0524 (13)	0.000 (2)	−0.0004 (12)	−0.0025 (14)
C1	0.0535 (15)	0.0875 (18)	0.0549 (14)	−0.0038 (18)	0.0029 (15)	−0.0151 (14)
C2	0.0379 (11)	0.098 (2)	0.0413 (12)	0.0055 (18)	−0.0016 (9)	−0.0081 (13)
C3	0.105 (3)	0.097 (2)	0.0711 (19)	0.008 (3)	0.006 (2)	0.0113 (17)
C4	0.0316 (10)	0.0561 (14)	0.0484 (12)	0.0012 (13)	−0.0022 (9)	−0.0071 (11)
C5	0.0297 (11)	0.0698 (16)	0.0504 (13)	0.0019 (14)	−0.0029 (9)	−0.0091 (12)
C6	0.0322 (10)	0.0680 (14)	0.0460 (11)	−0.0005 (15)	−0.0020 (9)	−0.0014 (12)

### Geometric parameters (Å, °)

**Table:**

O1—C1	1.396 (3)	C1—H1A	0.9700
O1—H1C	0.8200	C1—H1B	0.9700
O2—C4	1.215 (3)	C2—C3	1.509 (5)
O3—C5	1.213 (3)	C2—H2A	0.9800
N1—C4	1.316 (3)	C3—H3A	0.9600
N1—C2	1.468 (2)	C3—H3B	0.9600
N1—H1D	0.8600	C3—H3C	0.9600
N2—N3	1.113 (3)	C4—C5	1.538 (3)
N2—C6	1.310 (3)	C5—C6	1.409 (3)
C1—C2	1.497 (4)	C6—H6A	0.9300
			
C1—O1—H1C	109.5	C3—C2—H2A	108.6
C4—N1—C2	124.25 (19)	C2—C3—H3A	109.5
C4—N1—H1D	117.9	C2—C3—H3B	109.5
C2—N1—H1D	117.9	H3A—C3—H3B	109.5
N3—N2—C6	178.0 (3)	C2—C3—H3C	109.5
O1—C1—C2	113.2 (3)	H3A—C3—H3C	109.5
O1—C1—H1A	108.9	H3B—C3—H3C	109.5
C2—C1—H1A	108.9	O2—C4—N1	125.6 (2)
O1—C1—H1B	108.9	O2—C4—C5	120.4 (2)
C2—C1—H1B	108.9	N1—C4—C5	114.01 (18)
H1A—C1—H1B	107.7	O3—C5—C6	125.0 (2)
N1—C2—C1	107.5 (2)	O3—C5—C4	121.1 (2)
N1—C2—C3	110.9 (3)	C6—C5—C4	113.92 (18)
C1—C2—C3	112.6 (2)	N2—C6—C5	116.8 (2)
N1—C2—H2A	108.6	N2—C6—H6A	121.6
C1—C2—H2A	108.6	C5—C6—H6A	121.6
			
C4—N1—C2—C1	−112.0 (3)	O2—C4—C5—O3	−178.9 (4)
C4—N1—C2—C3	124.6 (3)	N1—C4—C5—O3	1.3 (5)
O1—C1—C2—N1	176.3 (3)	O2—C4—C5—C6	0.4 (5)
O1—C1—C2—C3	−61.3 (3)	N1—C4—C5—C6	−179.5 (3)
C2—N1—C4—O2	−5.2 (5)	O3—C5—C6—N2	−2.7 (5)
C2—N1—C4—C5	174.6 (3)	C4—C5—C6—N2	178.1 (2)

### Hydrogen-bond geometry (Å, °)

**Table:**

*D*—H···*A*	*D*—H	H···*A*	*D*···*A*	*D*—H···*A*
O1—H1C···O1^i^	0.82	1.99	2.811 (4)	179.
N1—H1D···O2^ii^	0.86	2.35	3.1024 (18)	146.
C6—H6A···O3^iii^	0.93	2.18	3.039 (3)	153

Symmetry codes: (i) *x*+1/2, −*y*+1/2, −*z*; (ii) *x*+1, *y*, *z*; (iii) *x*−1, *y*, *z*.
